# Perceived benefits, challenges, and recommendations for HIV research dissemination and implementation science efforts in Tanzania: Findings from the HIV/AIDS Research Forum brainstorming session

**DOI:** 10.1371/journal.pgph.0000952

**Published:** 2022-10-26

**Authors:** Donaldson F. Conserve, Subina Saini, Jumanne Issango, Andrew M. Kilale, Jerome Kamwela, Leonard Maboko, Wynton Sims, Sylvia Shirima, Julianna Vargo, Ryleigh Rawson, Abigail Ondrus, Echezona E. Ezeanolue, Guy-Lucien Whembolua

**Affiliations:** 1 Department of Prevention and Community Health, Milken Institute School of Public Health, The George Washington University, Washington, District of Columbia, United States of America; 2 Department of Health Behavior, Gillings School of Global Public Health, University of North Carolina at Chapel Hill, Chapel Hill, North Carolina, United States of America; 3 College of Medicine, Medical University of South Carolina, Charleston, South Carolina, United States of America; 4 Tanzania Commission for AIDS (TACAIDS), Dar es Salaam, Tanzania; 5 National Institute of Medical Research, Muhimbili Medical Research Centre, Dar Es Salaam, Tanzania; 6 San Francisco School of Medicine, University of California, San Francisco, California, United States of America; 7 Arnold School of Public Health, University of South Carolina, Columbia, South Carolina, United States of America; 8 Center for Translation and Implementation Research, College of Medicine, University of Nigeria, Nsukka, Nigeria; 9 HealthySunrise Foundation, Las Vegas, Nevada, United States of America; 10 Department of Africana Studies, University of Cincinnati, Cincinnati, Ohio, United States of America; University of California San Francisco, UNITED STATES

## Abstract

Although several international and national HIV/AIDS conferences exist, there was not a national conference in Tanzania focusing on HIV/AIDS disseminating and implementation research conducted in the country and abroad. This created a missed opportunity for researchers to share their research findings with local policymakers and HIV program implementers who can influence the adoption and implementation of promising research in public health and clinical practice settings. In response, the first HIV/AIDS D&I Research Forum designed to enhance local D&I efforts for HIV research, was organized in Tanzania in 2018. This paper explores the perceived benefits of the HIV/AIDS D&I Research Forum and potential challenges of developing similar forums and recommendation for future HIV research D&I conference in Tanzania. During the second day of the Forum, which was held in September 2018 in Morogoro, Tanzania, a 1-hour structured brainstorming session was conducted with the Forum attendees (n = 50), including researchers, medical professionals, policymakers, representatives from different ministries. Transcription of the brainstorming session was analyzed to identify benefits of the Forum, perceived challenges for organizing similar HIV/AIDS research dissemination events, and recommendations for addressing the challenges. Overall, participants perceived the forum to be beneficial because it provided opportunities for strategic collaborations between researchers, policymakers, and other stakeholders and for them to discuss challenges for D&I efforts. Forum attendees also identified several potential challenges for future D&I research forums such as the abstract requirement which may deter non-researchers, costs, meeting frequencies, and lack of funding and coordination between organizations involved in D&I research efforts. To address these concerns, a recommendation was made to host a biennial national conference in order to allow more time for ethical review and feedback that can enhance contribution of the project to D&I efforts and to raise funds. The benefits identified for the Forum highlight the importance of organizing similar D&I meetings for HIV-related research at the national level in Tanzania. However, the potential challenges discussed need to be addressed in order to develop a sustainable national D&I research conference by incorporating recommendations that forum attendees proposed.

## Introduction

Although significant progress has been made to address the burden of HIV/AIDS, it remains a pressing public health issue in Tanzania [1]. In Tanzania, only 960,000 people were aware of their HIV status and an estimated 1.4 million people are living with HIV [2]. The significant social and economic impacts of the epidemic can be observed in the loss of human capital and the damaging effects on the country’s institutional capacity [1]. In response, international and local stakeholders, including the Tanzania Commission for AIDS (TACAIDS), and its partners monitor trends, risk factors, and the prevalence of HIV infection, alongside initiatives to guide strategic planning in the fight against HIV/AIDS [2, 3].

TACAIDS is a government organization established by the late President Benjamin Mkapa in 2000 to coordinate the efforts of stakeholders in the multi-sectoral response to HIV/AIDS in Tanzania [3]. In 2001, TACAIDS developed the national policy on HIV/AIDS [2, 3]. The National Multisectoral Strategic Framework was developed to organize and coordinate the contributions of stakeholders to the HIV/AIDS response in Tanzania and to address the damaging and nationwide impact of the HIV/AIDS epidemic [3]. Since its establishment, the organization has taken a strategic leadership role in communicating information about HIV/AIDS. TACAIDS collaborates with several domestic and international partners such as the National AIDS Control Programme (NACP) and the United States Agency for International Development (USAID) to disseminate comprehensive population and health surveys, and implements programs to combat the damaging social, economic, and health-related effects of HIV/AIDS [2, 3]. For example, TACAIDS, under the leadership of the Prime Minister, in collaboration with the Benjamin Mkapa Foundation, and the National Institute for Medical Research at Muhimbili Medical Research Centre developed the 2018–2020 Male Catch-Up Plan [4] to guide implementation research [5–9] and national programs to increase HIV testing services uptake among men in Tanzania. In addition, TACAIDS has supported the formative research needed to disseminate and implement HIV self-testing interventions and programs in Tanzania [7].

In an effort to enhance the local dissemination and implementation of HIV-related research in Tanzania, TACAIDS organized the first HIV/AIDS Research Forum, which was held on September 2018 in Morogoro, Tanzania. There has been an increase in the interest for dissemination and implementation (D&I) science with institutions such as the National Institutes of Health (NIH) providing support through research fundings, conferences, and workshops [10] similar to the HIV/AIDS Research Forum organized by TACAIDS. For example, the NIH has organized several meetings to support the advancement of the science of D&I [11]. One of the most notable ongoing D&I meetings is the annual Conference on the Science of Dissemination and Implementation [12]. In addition, several other institutions and international associations have supported HIV-related D&I science efforts such as the Adolescent HIV Prevention and Treatment Implementation Science Alliance and international HIV conferences [13–20].

Dissemination is defined as an active approach of spreading evidence-based-interventions (EBIs) to non-researchers such as policymakers, clinicians, and public health practitioners using different engagement strategies [21, 22]. The goal of the science of D&I is to address the barriers that prevent the translation of research findings into clinical and public health practices [22]. However, prior research has found dissemination efforts among academics to be poor and identified numerous barriers to dissemination of EBIs by academics to non-research audience, which hinder the implementation of research findings into practice [23, 24]. The barriers for dissemination include, but are not limited to, lack of practice experience among academics, limited support and funds for researchers who might want to disseminate their research findings [23–25]. Local face-to-face meetings with stakeholders such as policymakers, clinical service providers, and community members are crucial because researchers rated these meetings as the most impactful for dissemination efforts and translation of research findings into practice and policy [26]. In addition, guidelines for dissemination efforts developed by the Community Advisory Board of the University of California, San Francisco, Center for AIDS Prevention Studies strongly support the dissemination of research findings by researchers to policymakers and other non-researchers through different forums [27].

Although a variety of international and national HIV/AIDS conferences exist [13–19], there was no nationally coordinated effort designed to organize meetings for dissemination of HIV-related research in Tanzania before the first HIV/AIDS D&I Research Forum. Similar to the meetings organized by the NIH and other organizations to support researchers and other stakeholders interested in D&I efforts [11], TACAIDS organized the HIV/AIDS D&I Research Forum to support researchers and representatives from various organizations who are interested in D&I for HIV-related research. The objectives of the Forum were to: 1) disseminate HIV research findings and exchange of knowledge and generate insights and challenges into issues facing HIV research; 2) document key research findings related to policy direction and decision-making and; 3) discuss next steps for intervention implementation of EBIs for and HIV prevention, treatment, and care in the country.

The aim of this paper is to inform the development of future D&I science efforts related to HIV in Tanzania using a brainstorming session approach that was conducted with attendees of the HIV/AIDS D&I Research Forum. The brainstorming session was conducted to explore: 1) the perceived benefits of the HIV/AIDS D&I Research Forum; 2) potential challenges of developing similar forums and; 3) recommendation for organizing future HIV/AIDS D&I Research Forums.

## Materials and methods

### Setting and participants

A two-day HIV/AIDS D&I Research Forum was organized by TACAIDS in Morogoro, a town in eastern Tanzania, at Sokoine University of Agriculture on September 27-28^th^, 2018. A total of 50 attendees were at the forum representing ministries and institutions with diverse background and specialties, including but not limited to, academicians, researchers, policy and decision makers, funding agencies, and implementation partners. Fifteen presentations were made that varied from the 2016–2017 Tanzania HIV Impact Survey (THIS), to the assessment of the impact of behavior change communication strategies on sexual behaviors among youth, overview of HIV vaccine trials in Tanzania, lessons learned from supporting adolescents living with HIV in Tanzania, and HIV/AIDS coordination mechanism in resource-scarce settings in Tanzania.

### Design and procedures

The project design consisted of a brainstorming session followed by an exploratory analysis of the qualitative data from the session. Brainstorming has been used in other studies and several recommendations for how to use the technique have been reported [28]. To prepare for the brainstorming session, the team members met (DFC, JI, AMK) prior to the forum to develop the discussion guide based on the literature on similar brainstorming sessions that have occurred with program implementing partners, researchers, and policymakers at different D&I conferences. In particular, the discussion guide was informed by the brainstorming session and group structured activities conducted by co-author (EE) with stakeholders who attended the first national conference organized by the Nigerian Implementation Science Alliance to assess barriers and research gaps related to sustainable interventions and implementation strategies related to HIV prevention, care, and treatment [29]. Building on this research, the academic- practice partnership [30], and prior research projects [4, 7] between TACAIDS and researchers at the National Institute of Medical Research in Tanzania and different universities in the United States, the discussion guide was developed in consultation with TACAIDS’s leadership for review, suggestions, and approval before the forum.

The epistemological and ontological assumptions of the brainstorming session are based on pragmatism which posits that knowledge may be discovered by examining the usefulness of theory in practice [31]. Because pragmatism sees knowledge as contextually contingent, a group discussion, such as the one used in this study, can provide valuable information regarding central topics for which stakeholders might have different perceptions [31]. For pragmatists, reality is that which is practical [31]. Because knowledge is derived, in part, from analyzing the interaction among a group of individuals, the brainstorming session is a pragmatic way to activate a range of ideas regarding the perceived benefits of the HIV/AIDS D&I Research Forum as well as challenges of organizing future forums and recommendations for addressing these challenges. The consolidated criteria for reporting qualitative research (COREQ) checklist was used to ensure the reporting of the study procedures and findings are consistent with the guideline (S1 File) [32].

### Recruitment and data collection

On the second day of the forum, all the forum attendees were provided information about the brainstorming session by co-authors (LM, DFC, AMK) and given an opportunity to raise any questions and decide if they wanted to participate. All 50 forum attendees provided informed consent to participate and discuss the perceived benefits of the forum and recommendations for how to organize future D&I forums and a potential national D&I conference focusing on HIV-related research.

A brief reminder of the purpose of the HIV/AIDS D&I Research Forum was provided in the beginning of the brainstorming session. The following questions served as guiding points for the brainstorming discussion: 1) Is there an interest in holding similar HIV research meetings?; 2) How should future meetings be organized and how frequent?; 3) Should future meetings be held at the national, zonal, or regional level?; 4) What are potential challenges of organizing future meetings?; 5) What are the recommendations on how to overcome those challenges when organizing future meetings? The questions were developed for the brainstorming session. Oral instead of written consent was chosen due to the format of the HIV/AIDS D&I Research Forum. Oral consent for group members was documented by using the attendance sheet and asking each attendee to either confirm their consent or decline before the audio recording began for the brainstorming session. The brainstorming session lasted approximately 1 hour, and no compensation was provided beyond the per diem that all attendees received for attending the HIV/AIDS D&I Research Forum.

### Data analysis

The analysis was underpinned in a pragmatism methodological orientation and included a descriptive qualitative analysis [31]. The focus of the analysis was to understandthe perceived benefits of the HIV/AIDS D&I Research Forum as well as challenges of organizing future forums and recommendations for addressing these challenges. The research assistants (SS, JV, RR, AO) transcribed the audio-recording of the brainstorming session and reviewed the transcript while listening to the audio-recording multiple to verify the transcribed data were accurate. After the transcription phase, the experienced team member (DFC) provided training in qualitative data analysis to the research assistantsin multiple one-hour session over three weeks covering how to develop a codebook, apply codes to the data, identify inductive codes, and reporting the primary and sub-codes. The combination of deductive and inductive coding was informed by the Coding Manual for Qualitative Researchers [33]. Deductive codes were used namely perceived benefits, challenges, and recommendations which were in a previous qualitative research conducted in partnership with TACAIDS on HIV self-testing in Tanzania [7], with primary and sub-code definitions and quotes supporting these codes [34]. SS, JV, RR, and AO coded the transcripts in analytical phases by applying deductive codes but also identifying emerging codes. Deductive codes are a priori codes selected from the literature review and our prior research whereas the emerging codes are inductive codes that came from the specific responses from the attendees. Coders reviewed the data and applied these structural codes with at least two research assistants coding a subset of the transcript and comparing their respective codes during consensus-coding meetings to resolve any discrepancies. In the second phase, single code reports were generated and reviewed before refining the codes and creating sub-codes related to the deductive codes which included: 1) perceived benefits of hosting HIV/AIDS D&I Research Forum; 2) perceived challenges of hosting similar forums; and 3) recommendations for overcoming these challenges. Inductive subcodes related to each primary deductive code were reviewed and assessed to create primary categories, which were used to structure the Results section. Quotations for each subcode were reviewed and grouped to better understand and demonstrate the range of content addressing the primary deductive code in question. A positionality memo was generated to track the researcher’s (DFC’s) identity in relation to the topic and the data and to help ensure that the coding process did not inaccurately impose topics on the data. The research assistants met regularly with the experienced team member (DFC) to ensure analytic rigor and establish agreement regarding code definitions, code application, and sub-codes. This analytic rigor helped to ensure trustworthiness of our coding and final analysis.

### Ethical considerations

Ethical approval for the brainstorming session was not required by the University of South Carolina Office of Research Compliance and the National Institute of Medical Research ethics committee in Tanzania. During the brainstorming session, attendees were informed that the information they shared may inform future research activities. Attendees were asked if they had any objection and all attendees provided verbal consent which was recorded.

## Results

There were 50 stakeholders who attended the two-day HIV/AIDS D&I Research Forum from different organizations and institutions, including TACAIDS and several ministries: 1) Ministry of Education, Science, and Technology; 2) Ministry of Finance and Planning, and; 3) Ministry of Energy. Other institutions represented were the United Nations International Children’s Emergency Fund (UNICEF), the National Institute for Medical Research, and Tanzania Center for Diseases Prevention and Control (CDC), National Bureau of Statistic (NBS). Attendees from academic and research institutions were from Sokoine University of Agriculture, Muhimbili University of Health and Allied Sciences (MUHAS), Catholic University Health and Allied Sciences (CUHAS), University of Dar es Salaam (USDM), University of South Carolina (USC), Henry M. Jackson Foundation Medical Research International (HJFMRI). The Morogoro Regional Commissioner and Regional Health Management Team were also in attendance.

### Perceived benefits of HIV/AIDS D&I Research Forum

One of the primary topics that emerged from analyzing the content coded to perceived benefits of hosting HIV/AIDS D&I Research Forum was the *opportunity for strategic collaboration among different stakeholders*. The gathering of policymakers from different ministries, researchers, programmers, and implementers allowed researchers to share their research findings and work in progress. It also created an opportunity to establish new collaborations and for non-researchers, policymakers, and other stakeholders who have the power to influence the policies, which is needed to support D&I efforts that can lead to the translation of research findings into policies and routine healthcare services:

The bad thing in our conferences that we’re making, we are just sitting on our own. There are no policymakers. There are no people who can decide in those conferences we are making. So, we are there discussing our findings. Off we go, nothing can be done. The good thing I’ve seen here [at the HIV/AIDS D&I Research Forum] is that we have the MP—Member Parliament…. We can bring the findings as scientists but, there are people who have no findings… They have power. We want them here!

So, this forum is a very important forum as we have different people [policymakers, non-researchers]. So, the thing that we can do now, just from TACAIDS is to see the key people from this group.

There are other group here I know who have never [attended] those conferences but through this [forum] they have a chance to present.

One participant also emphasized the importance of convening researchers who are leading research projects to discuss how they can collaborate instead of working separately for the same goal:

There are other people who are doing, uh, clinical research is like we are killing a snake. Someone is hitting the head, someone is hitting the tail. And, they can all decide to kill the snake depending on the intensity of hitting. So, what we can do right now is to, is like to, um, to certify that there are people who are doing clinical research, there are people who are doing social research… If we group these people and we make use of them and we coordinate all of them together and we plan on how we can meet and discuss.

Another benefit of the forum was that participants also raised *challenges for current EBIs being implemented* and *challenges for hosting similar HIV/AIDS D&I Research Forum* for which they eventually provided recommendations. For example, participants from different ministries, regions, and sectors were able to learn about the 2016–2017 THIS from a presentation made by a statistician from NBS. Given that the HIV Test and Treat policy was implemented in Tanzania since 2016 based on the global evidence for initiating ART early and the low HIV testing among men, participants raised the point that the lack of success with reaching men may be the fact that not all stakeholders from different sectors are involved in the implementation of the EBIs for HIV services targeting men and the need to engage all stakeholders:

But, next to that, one of the issues that happened clearly, even in this meeting we have seen, testing services are there. Drugs are there. But why do, especially men, not utilize our services? You need everybody else…

Even if I don’t get p-values, it’s not a big deal. But what are the real issues in the field? That’s why I want to bring implementers there. To have an opportunity to share their experiences… It’s not that one does not care for science and science can inform implementation. But implementation also has its own challenges. You have all what you think can work. But you go to the community. Things aren’t working.

### Perceived challenges for future HIV/AIDS D&I Research Forum

Aside from the perceived benefits, participants also reported potential challenges of organizing future D&I forum such as *research abstract requirement that may deter non-researchers needed* *for D&I efforts*. Since many research meetings require attendees to submit abstracts, one participant pointed out that many non-researchers such as policymakers and politicians who have great ideas that can support D&I efforts for HIV may be deterred from attending.

I don’t think that you have ever got something from the ministry over here, because they don’t write abstracts. Somebody cannot [may not] be a scientific but can have great ideas. We all see the politicians, we never have a paper from the parliament, but you [they] have great ideas, of which they are relating in this forum.

Another set of perceived challenges were reported related to the *meeting’s frequency*, *costs*, *& lack of coordination*. Participants shared that if the conference is held too frequently (i.e. annually), then there is the possibility that there will be repetitive presentations from year to year. This concern stemmed from the fact that researchers may not have enough time to complete their projects and advance to new projects within one year since research projects take a long time to be developed, funded, approved by institutional review boards, and carried out.

I was thinking, while I was there, the management we are discussing to, to make it two-years event. Because the annual rate, that even as scientists, we don’t have enough, enough results to come and present… So, the plan, I don’t know how far you’ve gone, but the plan was to make it a two-years event. You should know that to give room for scientists to do their research and come up with very tangible data. Otherwise, we are coming with preliminary results, coming to present.

Other potential challenges were related to the costs associated with organizing a national or regional HIV/AIDS conference, the lack of coordination between organizations (i.e. NACP and TACAIDS) involved in HIV/AIDS programs, and the potential time conflict between different HIV/AIDS conferences as demonstrated in the following quotes:

In addition to that, it has to be after every two years. After every two years because this is costly, you cannot do it on an annual basis.

I am now seeing the contradiction because we have NACP and we have TACAIDS, when you go to NACP they say they have the data, TACAIDS you have the implementations. So, you now think of organizing the events, the annual events. So, do you have any venue, that you can meet with NACP and TACAIDS. Then you think of actually organizing the same event. That’s one. Then, how about if you have so many events to organize, you have so many findings to disseminate.

### Recommendations for HIV/AIDS D&I Research Forum

One of the recommendations was to *hosting biennial meetings to allow enough time for review and feedback that can enhance D&I efforts*. Participants offered several suggestions for overcoming potential challenges described above and made key recommendations that can inform the development of future HIV/AIDS D&I Research Forum to support D&I research efforts in the country. First, participants agreed that there is a need to have these forums and some participants suggested such forums happen every two years to provide researchers more time for their projects to be reviewed and received feedback from the national ethics committee and other stakeholders to ensure the project is conducted thoroughly and increase the chances their results can be significant for translation into policies and routine healthcare practices:

I think we need them every, after every two years because we need researchers that will be really informative and that can be translated into policies. So, in coordinating these researchers, I am suggesting whoever is interested in researching on HIV/AIDS in this country, after getting a clearance, or before getting clearance from the National Institute for Medical Research…should get some input on the topic or on the title from TACAIDS, so that in the process of whatever you are doing, or you are researching on, TACAIDS should be expecting something out of it. So that at the end, this research can have the flavor or tastes of a policy that is needed in order to, to inform whatever interventions we are designing in the fight against HIV/AIDS. That is what I would like to put forward.

Second, participants recommended *increasing coordination between organizations*, *stakeholders and accessibility of scientific presentations*. For example, participants suggested that TACAIDS develop strategies in collaboration with other organizations to increase coordination, attract multi-sectoral stakeholders, and increase teamwork and inter-organizational communication.

I’m proposing you put up a task force so that you resolve everything that may [be] part of the contradictions. That way when we start moving, we move as a team, instead of moving as TACAIDS alone. Because in a way you go there to organize the events now that you need to organize.

Another related recommendation included greater cross-collaborations between researchers, policymakers, and program implementers prior to the conference in order to enhance the success of the conference and the implementation of ongoing and future EBIs for different groups.

Regarding this recommendation, participants also stated that future forums and conferences should allot the first day for scientific presentations and the second day for policy dialogue and action plans.

We make engagements of policy and decision makers. Because for me, we should have been engaging for the past fifteen years but we just sit there ourselves, on a committee ourselves… We could say maybe, the first day, for the scientific but the other day is the policy dialogue, engagement and the accommodations, actions to make change. And That’s really a good direction, I think that’s a very good reason. Because we are scientist but uh how would we engage them to make things happen?

Participants also stated that presentations from personnel who do not submit scientific papers but can share relevant ideas. Furthermore, discussion-based forums, where presentations are delivered in simple language instead of in complex scientific terms and statistics, are preferred in order for members outside of the scientific community to understand.

I think I’ve been into research, and sometimes we struggle even to package, to package, what findings can change policy and where policy breeds them and whatever. Just make it simple for people to understand.

I just want to say something that will strengthen what has already been said. Yes, how the meetings should be organized. I’m happy we now are moving away from p values and confidence intervals. We are going to the language which is understood by the community.

Because you say you are mandated to disseminate this information. There is a way maybe you are talking forth. Can they digest it, the findings from different, maybe for clinical or social findings and for something, and come up with something that can inform policy makers as a tool at the end of the day? We could even disseminate it to the community they can access books that they can read, even in schools they can access all the information in a simple way, not in a scientific way. In a simple way that our community can understand our findings or our presentation.

## Discussion

To our knowledge, this is the first study designed to explore the perceived benefits of an HIV/AIDS D&I Resarch Forum in Tanzania, assess potential challenges associated with organizing similar forums, and seek recommendations on how to develop future forums. The main perceived benefit of the forum was the promotion of collaborative relationships among stakeholders from different sectors. A related benefit was also providing the non-academic space recommended [27] for researchers to disseminate research findings to policymakers, practitioners, and healthcare providers who can translate research findings into policies and adoption of interventions to public health and clinical practice settings [11, 35]. This finding is also supported by a study conducted in Bostwana which found that the inclusion of high-level government representatives from different ministries in the Madikwe Forum has fostered a platform for informed discussion about HIV-related programming and implementation that have been helpful in reducing bottlenecks that can prevent timely and effective implementation of projects [36]. Another benefit of the forum and brainstorming session was that participants also had a chance to discuss challenges of planning activities such as the HIV research forum in Tanzania to support the dissemination and implementation of EBIs for HIV prevention, care, and treatment.

Similarly, other forums such as the meetings organized by the Nigerian Implementation Science Alliance have provided stakeholders an opportunity to identity challenges of implementing EBIs for HIV prevention programs [29]. The challenges reported by stakeholders at the forum such as the lack of coordination between government, program implementers, and researchers parallel some of the obstacles identified at the D&I forum for prevention of mother-to-child transmission of HIV programs in Nigeria [29]. The need for D&I forums similar to the HIV/AIDS Research Forum is supported by the positive outcomes from prior but smaller meetings that were organized by TACAIDS to address low HIV services uptake among men. For example, TACAIDS organized smaller D&I meetings to address the challenge of reaching men and these gatherings resulted in the development of the Male Catch-Up Plan [4], which consisted of different EBIs such as HIV self-testing that have since been implemented to reach men with the support of policymakers, implementing partners, and donors in Tanzania and other sub-Sahara African countries [37–39].

One of the objectives of organizing larger meetings such as the HIV/AIDS D&I Research Forum is to provide different stakeholders an opportunity to collaborate and identify challenges preventing the implementation of EBIs for different hard-to-reach groups in Tanzania and develop strategies based on existing EBIs and local research conducted in Tanzania that can be implemented and scaled up similar to the Male Catch-Up Plan [4]. However, stakeholders raised a number of concerns for such forums such as the potential poor attendance of policymakers and other non-researchers because of the abstract requirements for the D&I forums. Poor attendance of high-level representatives has been observed at other forums such as the Madikwe Forum in Bostwatna [36]. The potential poor attendance of policymakers and different ministries is a valid concern, especially for researchers, since policymakers have the decision-making power but are not usually present in research meetings. Recommendations to enhance attendance from policymakers and non-researchers included the removal of the abstract requirement. Another recommendation that can improve attendance for similar forums in Tanzania and other low middle-income countries (LMICs) is having the meeting less frequently such as every two years, which supports the findings from the Madikwe Forum in Bostwatna [36].

### Limitations

Despite the strengths of the findings, there are some limitations worth highlighting. The challenges and recommendations do not represent the views of several stakeholders and organizations involved in HIV/AIDS research D&I that were not present at the forum. In addition, the majority of the project team members are implementing partners, researchers and not policymakers. Therefore, the interpretation of the results may be influenced by the team members’ background. Third, the brainstorming session might have privileged individuals in more authoritative roles than those who are not and attendees with less positional power might have felt less inclined to share their views because of fear of judgment. Fourth, the brainstorming session could have led to socially desirable and thereby inauthentic comments. The other limitation was that the recording from the brainstorming session proved to be difficult to decipher at times. To address this issue, multiple members of the team listened and re-transcribe segments of the audio that were difficult to decipher.

### Implications for practice

Stakeholders made key recommendations to address the potential challenges for D&I efforts for HIV and other diseases that have clear implications for practice. First, there is a need to develop strategies that can attract non-researchers to attend D&I research meetings. Given that the main perceived benefit of the forum was the opportunity for stakeholders, especially non-researchers, from different sectors to meet and collaborate with each other for D&I efforts, TACAIDS and related organizations must design new strategies to remove barriers such as abstracts or communicating to non-researchers that their attendance and voices are needed for the success of D&I and ongoing EBIs in the country. Second, stakeholders need to be engaged prior and beyond the meetings to sustain ongoing partnerships and contribute to the success of D&I efforts in the country. Third, organizations involved in D&I efforts for HIV must collaborate and resolve any issues in order to develop the leadership needed to lead D&I efforts for HIV and raise the funds needed to support future D&I meetings. Future HIV/AIDS research forums organized in Tanzania or in other LMICs should consider the benefits, challenges and recommendations identified in this study in order to support the development of similar forums that can support D&I research efforts for different key populations and hard-to-reach groups.

## Conclusions

The brainstorming session of the HIV/AIDS D&I Research Forum helped to identify several perceived benefits and challenges of the first HIV/AIDS Research Forum in Tanzania. The benefits identified included the promotion of collaborative relationships among individuals from different sectors and discussing the potential challenges of having future national HIV/AIDS meetings to support D&I research efforts in the country.

## Supporting information

S1 File COREQ checklist. (DOCX)
