# Supplementary material for: Perceived benefits, challenges, and recommendations for HIV research dissemination and implementation science efforts in Tanzania: Findings from the HIV/AIDS Research Forum brainstorming session
Source: PLOS Glob Public Health. 2022 Oct 26;2(10):e0000952. doi: 10.1371/journal.pgph.0000952 (PMC10022396; doi:10.1371/journal.pgph.0000952)
Supplement: S1 File — (DOCX) [file pgph.0000952.s001.docx]

**COREQ Table for Perceived Benefits, Challenges, and Recommendations for HIV Research Dissemination and Implementation Science Efforts in Tanzania: Findings from the HIV/AIDS Research Forum Brainstorming Session**

| Personal Characteristics 1. Interviewer/facilitator, 2. Credential, 3. Occupation, 4. Gender, 5. Experience and training | The brainstorming session was conducted by co-authors (DFC, AMK) who are researchers with over ten years of experience conducting quantitative and qualitative data collection. DFC and AMK were trained in the social and behavioral sciences and each has a PhD. DFC is an associate professor at the George Washington University and AMK is a senior researcher at the National Institute of Medical Research at Muhimbili University of Health and Allied Sciences. |
| --- | --- |
| Relationship with participants 6. Relationship established, 7. Participant knowledge of interviewer, 8. Interviewer characteristics | DFC did not know the participants prior to the brainstorming session. However, AMK and the TACAIDS director (LM) knew some of the Forum participants. To reduce social desirability bias, participants were informed to only mention the name of the institution they work without including their names. |
| Theoretical framework 9. Methodological orientation and Theory | The study was underpinned in a pragmatism methodological orientation and included a descriptive qualitative analysis [27] aimed at understanding perceived benefits of the Forum as well as challenges of organizing future Forums and recommendations for addressing these challenges. |
| Participant selection 10. Sampling, 11. Method of approach, 12. Sample size, 13. Non-participation | A purposive sampling approach was used to recruit Forum participants who were well equipped to provide expertise on the perceived benefits of the Forum and recommendations for how to organize future Forums and a national conference focusing on HIV/AIDS. A total of 50 attendees were at the Forum representing ministries and institutions with diverse background and specialties. All attendees provided verbal consent and agreed to participate in the brainstorming session. |
| Setting 14. Setting of data collection, 15. Presence of non-participants, 16. Description of sample | The brainstorming session was conducted in September 2018 during the HIV/AIDS Forum in Morogoro, a town in eastern Tanzania. The Forum was held at Sokoine University of Agriculture on September 27-28^th^. A description of the institutions the participants from the brainstorming session came from are provided in the results section. |
| Data collection 17. Interview guide, 18. Repeat interviews, 19. Audio/visual recording, 20. Field notes, 21. Duration, 22. Data saturation, 23. Transcripts | There were no repeat interviews. The notes taken during the brainstorming session were compared with the audio-recording to support or clarify some statements in the transcript. The brainstorming session lasted approximately an hour. We did not take a data saturation approach, and therefore the brainstorming session lasted to obtain the widest range of possible views and experiences. |
| Data analysis 24. Number of data coders, 25. Description of the coding tree, 26. Derivation of themes, 27. Software, 28. Participant checking | SS, JV, RR, and AO coded the transcripts in analytical phases by applying deductive codes but also identifying emerging codes. Coders reviewed the data and applied these structural (deductive) codes. Reliability and validity were established by having at least two research assistants code a subset of the transcript and comparing their respective codes during consensus-coding meetings to resolve any discrepancies [29]. The analysis was not based on constructing themes. Rather, it focused on identifying primary topics in a descriptive manner. |
| Reporting 29. Quotations presented, 30. Data and findings consistent, 31. Clarity of major themes, 32. Clarity of minor themes | We report the results using illustrative quotes only for major categories. Consistency between the data and findings presented was checked as part of the iterative cycles of constant comparison in our analysis between the audio-recording, transcript, and notes. |
